# Is Cervical Stabilization Exercise Immediately Effective in Patients with Chronic Neck Pain and Upper Cervical Spine Dysfunction? Randomized Controlled Trial

**DOI:** 10.3390/life12050714

**Published:** 2022-05-11

**Authors:** Jacobo Rodríguez-Sanz, Miguel Malo-Urriés, María Orosia Lucha-López, Jaime Corral-de-Toro, Vanessa González-Rueda, Carlos López-de-Celis, Albert Pérez-Bellmunt, César Hidalgo-García

**Affiliations:** 1 Faculty of Medicine and Health Sciences, Universitat Internacional de Catalunya, 08195 Barcelona, Spain; vgonzalez@uic.es (V.G.-R.); carlesldc@uic.es (C.L.-d.-C.); aperez@uic.es (A.P.-B.); 2 ACTIUM Anatomy Group, Universitat Internacional de Catalunya, 08195 Barcelona, Spain; 3 Faculty of Health Sciences, Universidad de Zaragoza, 50009 Zaragoza, Spain; malom@unizar.es (M.M.-U.); 682825@unizar.es (J.C.-d.-T.); hidalgo@unizar.es (C.H.-G.); 4 Physiotherapy Research Unit, Universidad de Zaragoza, 50009 Zaragoza, Spain; 5 Fundació Institut Universitari per a la Recerca a L’ATENCIÓ Primària de Salut Jordi Gol i Gurina, 08007 Barcelona, Spain

**Keywords:** spine, neck pain, manual therapy, exercise, orthopedic manipulation

## Abstract

Purpose: To compare the effectiveness of a single exercise session with manual therapy techniques in the segments of the upper cervical spine (C0–1, C1–2 and C2–3), against a single exercise session in patients with chronic neck pain and mobility deficits in the upper cervical spine. Methods: A single-blind randomized controlled trial was performed. Fifty-eight patients were recruited (29 for the manual therapy and exercise group and 29 for the exercise group) who presented chronic neck pain and upper cervical spine dysfunction. The exercise focused on the deep muscles. The manual therapy combined manipulations and mobilizations with these exercises. Cervical range of motion, flexion-rotation test, pressure pain threshold and pain intensity were measured by a blind evaluator before and after the intervention. Results: Compared to pre-intervention, after intervention, the exercise group was significantly lower in terms of the range of motion, flexion-rotation test, and pressure pain threshold (*p* < 0.05). The manual therapy and exercise group improved in upper cervical flexion, the flexion-rotation test and intensity of pain (*p* < 0.05). Conclusions: It may be necessary to normalize the mobility of the upper cervical spine before cervical stabilization training, in patients with chronic neck pain and mobility deficits in the upper cervical spine.

## 1. Introduction

Chronic neck pain is described as pain located between the occiput and the third thoracic vertebra that persists for more than 3 months representing 14.6% of all musculoskeletal health problems [1]. Fifty percent of the adult population will experience cervical pain at some point during the year [2].

During rehabilitation in these patients, exercises were considered one of the most evidence-based modalities [3]. Specifically, spinal stabilization exercises have been used to activate the deep flexor muscles and decrease overactivity of the superficial muscles [4,5]. Systematic reviews and meta-analysis have shown that exercise therapy has beneficial clinical effects for most musculoskeletal conditions [6], including cervical pain over the medium and long term and even after the first treatment [7]. Moderate evidence supports the effects of cervico-scapulothoracic and upper extremity strengthening for pain reduction immediately post treatment [7]. Cranio-cervical flexion therapeutic exercises have shown to provide immediate pain relief, on movement in patients with chronic neck pain [8]. However, we do not know whether patients with upper cervical restriction can benefit from these exercises immediately.

In the rehabilitation process, some techniques additional to exercises may also be used to restore function and decrease pain. A useful method may be manual therapy (MT) to the soft tissues and joints to restore range of motion (ROM), decrease pain, and increase function. MT, including manipulation or mobilization, has been used to improve neck pain [5]. Moderate-to-high quality evidence has been reported previously regarding immediate improvements in pain intensity obtained from a single session of spinal manipulation in chronic mechanical neck pain in adults [9]. A single session of thoracic manipulation has been effective to increase flexion and rotation of the cervical region in adults with mechanical neck pain [10]. A single session of manipulation and range of motion exercises has been shown to increase scapulothoracic strength in subjects with neck pain [11].

The upper cervical spine is responsible for about 50% of total cervical rotation and for the “chin tuck” maneuver (consisting of a gentle glide of the chin straight back; with the chin being close to the throat, without moving the head up or down, bending the neck forward or holding the breath) used for exercising the deep neck flexor muscles, with a linear relationship being found between the degrees of upper cervical flexion and the ability to generate force with the deep flexor cervical muscles [12].

MT combined with various exercise approaches has been found to be superior to MT alone [5], but there is a lack of randomized clinical trials investigating MT in the segments of the upper cervical spine (C0–1, C1–2 and C2–3), in combination with stabilization exercises in patients with upper cervical spine dysfunction.

The hypothesis is that deep flexor cervical muscle stabilization exercises are effective in controlling symptoms of chronic cervical pain if they are performed with a physiological range of flexion of the upper cervical spine; otherwise, their effects may not be favorable.

The objective of this study is to compare the effectiveness of a single exercise session with manual therapy techniques in the segments of the upper cervical spine (C0–1, C1–2 and C2–3), against a single exercise session in patients with chronic neck pain and mobility deficits in the upper cervical spine.

## 2. Materials and Methods

### 2.1. Study Design

This was a single-blind randomized (simple 1:1) controlled trial of a larger study. The randomization process was carried out with the realization of a list of random numbers (1 and 2) created with the tool for randomization of numbers from the application Microsoft Excel 2010 (1 = manual therapy and exercise group; 2 = exercise group). To ensure random concealment of allocation, opaque envelopes (sealed and numbered) were prepared by one of the researchers not involved in the recruitment or the assessment of the patients.

The study was carried out in the “Delicias Sur” health center of Zaragoza, Spain (clinicaltrials.gov (accessed on 5 April 2022) number: NCT05085600). The ethical principles for research on human beings established in the Declaration of Helsinki, last revised in Fortaleza in October 2013, were complied with and approved by the local ethics committee (CEICA) with the “Acta Nº 13/2018” reference number.

### 2.2. Sample

The sample size was calculated based on the outcomes of Rodríguez-Sanz et al. (2021) [13]. The main variable used for sample size calculation was pressure pain threshold (PPT) obtaining a total sample of 58 patients (29 per group). The sample size was calculated using the GRANMO 7.12 program, with an α risk of 0.05, test two-side, a β risk of 0.20. We used an estimated common standard deviation of 106.79 kPa and a minimum expected difference of 88.26 kPa, estimating a follow-up loss of 20% [13].

**Figure 1:**
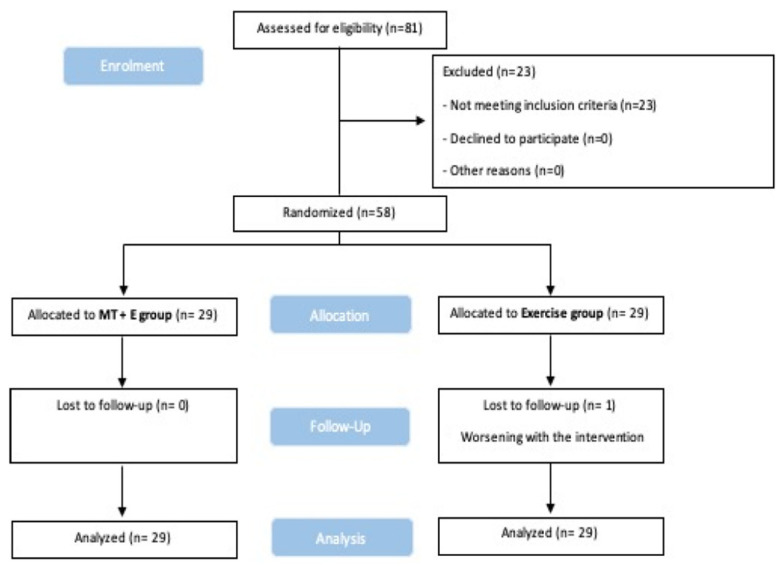
CONSORT. (Consolidated Standards of Reporting Trial) flow diagram.

Fifty-eight volunteers (17 male and 41 female), mean age 49.24 years (SD = 15.89), participated in the study. Inclusion criteria were patients with medical diagnosis of chronic idiopathic neck pain, after prior radiological examination, according to the International Association for the Study of Pain (IASP), as pain perceived anywhere in the posterior region of the cervical spine, from the superior nuchal line to the first thoracic spinous process [14], with more than 3 months of evolution [15], loss of mobility, through manual assessment, in the upper cervical spine segments (C0–1, C1–2 and C2–3) [16]. The manual assessment consisted of performing a traction on the segments looking for a decrease in the amount of opening and an increase in the resistance of the tissue towards the separation movement, with a reliability between 0.78 and 1 in cervical disorders [16,17]. A positive result in the flexion-rotation test (FRT) in the upper cervical spine (less than 32° or a difference of 10° or more between the two rotations) [18]. Not being able to exceed 24 mmHg in the cranio-cervical flexion test [19], being over 18 years old, and signing the informed consent. The exclusion criteria were contraindication to MT or exercise (pathological changes due to neoplasm, inflammation infections, osteopenia, or congenital collagenous compromise syndromes “Down’s, Ehlers-Danlos, Grisel, Morquio” [20]); marked degeneration of the cervical spine that may affect cervical spine ligament integrity [20]; history of trauma to cervical vessels and anticoagulant therapy or blood clotting disorders, or to have participated in any program of exercises or MT treatments designed to improve the performance of the cervical region in the previous 3 months; post-traumatic neck pain or red flags according to Rushton et al. [21]; an inability to maintain the supine position; use of pacemakers; an inability to perform the FRT; language difficulties that hinder understanding of informed consent or completion of the questionnaires necessary for this study; and patients with litigation or lawsuits pending (Figure 1).

An investigator with training and more than 10 years of experience performed the evaluation.

### 2.3. Measurements

The primary outcome measurements reported in this study were cervical PPT. Cervical mobility and intensity of pain were also used as secondary outcome measures.

Cervical PPT was measured using a digital algometer (Somedic AB Farsta, Somedic SenseLab AB, Sösdala, Sweden) with a round surface area of 1 cm^2^, and pressure was applied at the rate of 1 kg/cm^2^/s perpendicular to the skin. With the subject supine, PPT was assessed over 6 points bilaterally with a 10 s rest between each measurement: first metacarpal joint (MCJ), upper trapezius muscle, elevator of scapula, C5–6 zygapophyseal joint, C2–3 zygapophyseal joint and suboccipital muscles. Patients were instructed to press the button of the digital algometer at the precise moment that pressure sensation changed to pain. The mean of 3 trials was calculated over each point and used for analysis. PPT measurements have been found to have high reliability (intraclass correlation coefficient = 0.92–0.99) [22].

**Figure 2:**
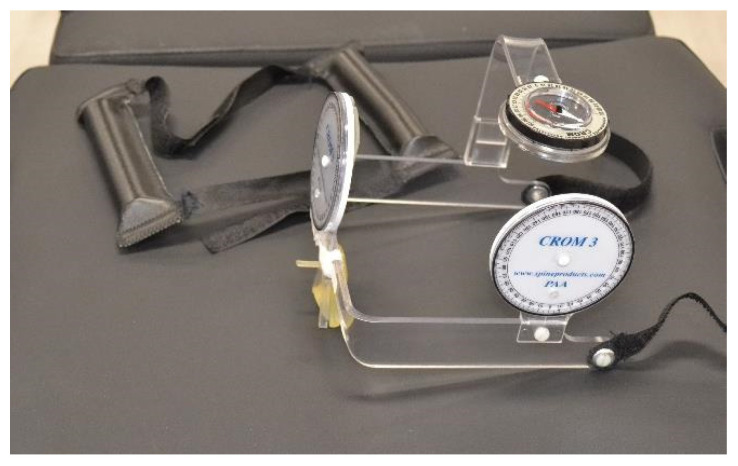
CROM device.

Active range of motion mobility was evaluated in a sitting position with the back vertical and resting on the backrest of the chair with a CROM device (floating compass; Plastimo Airguide, Inc., Buffalo Groove, IL, USA). The CROM measurement equipment is composed of a helmet-shaped structure with the shape of glasses. These glasses are adjustable to the nasal septum thanks to a Velcro located on the back to adjust them to the head. Three inclinometers are placed in the plastic structure to measure on the 3 cardinal planes. The frontal inclinometer, located in the medium front part of the structure, is a gravity inclinometer that is used to measure movements on the frontal plane, namely, the right and left cervical inclination. The sagittal inclinometer, located on the upper left side of the structure, is a gravity inclinometer that is used to measure movements on the sagittal plane, namely, cervical flexion and extension. One horizontal compass, located in the middle-upper part of the structure, is a magnetic compass that is used to measure movements on the horizontal plane, namely, right rotation and left rotation (Figure 2).

Flexion, extension, right and left side-bending and right and left rotation were measured. The range of motion in flexion and extension of the upper cervical region was measured in a standing position with the back against a wall [23]. Three measurements were made for each movement, and the result was the mean of the three measurements [24]. CROM measuring instrument has shown high intraexaminer reliability between 0.63 and 0.97 [25] and a high instrumental validity that has obtained correlation with radiographic measurements between 0.87 and 0.97 [26,27,28]. The measure inaccuracy of the CROM ranges has been established by one study as being between 5 and 10° [29].

FRT was used to measure the upper cervical rotation, predominantly at C1–2. To perform FRT, the patients were supine and the evaluator passively positioned the subject’s cervical spine to its maximum flexion and then rotated the head to the right and left side with the occiput resting against the evaluator’s abdomen. The movement stopped at whichever situation occurs first, either the subject presents symptoms, or the evaluator reaches the end of the range of motion and finds a firm end feel. A CROM device was used, and three measurements were performed for each movement, with the result being the mean of the three measurements [24].

The intensity of pain in the cervical region was measured with VAS from 0 to 100 mm in length, with the extremes defined as “no pain” (0) and “the worst pain imaginable” (100) and without any intermediate points. The test–retest reliability was observed to be excellent (ICC 0.92) [24].

An investigator with orthopedic MT specialist training and more than 10 years’ experience, performed the outcome measures before and after the intervention. The investigator was blind to the allocation group of each patient throughout the process.

### 2.4. Intervention

The treatment was applied by the same physical therapist with more than 10 years of MT experience.

#### 2.4.1. Exercise Group

**Figure 3:**
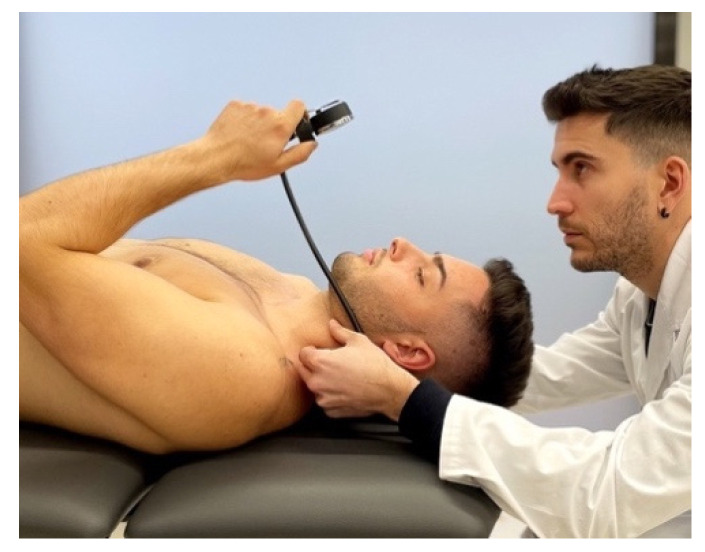
Contraction of deep neck flexor muscles.

After the baseline assessments, patients began performing the cervical stabilization exercise, and were taught to perform the contraction of deep neck flexor muscle activity with the help of the Stabilizer Pressure Biofeedback Unit (Chattanooga, TN, USA) in supine [30]. Exercises were undertaken in the supine position with the cervical spine in a neutral position [31]. Exercises were always carried out without pain, because pain can be an inhibitor of muscle contraction (Figure 3) [31]. Deep neck flexor muscle exercise consisted of a low load movement of the head to the inner range of cranio-cervical flexion [32].

The exercise group carried out one 20 min session, composed of 2 sets of 10 repetitions, holding each exercise for 10 s [31], a 40 s rest between each repetition and 2 min between blocks.

#### 2.4.2. Manual Therapy and Exercise Group (MT + E)

**Figure 4:**
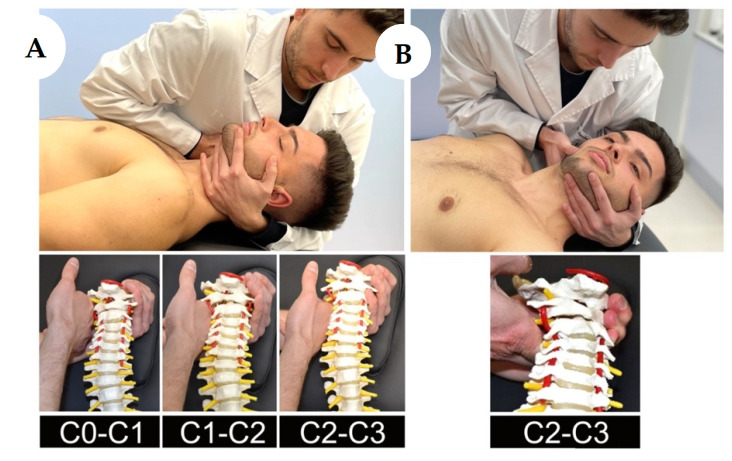
Manipulation Techniques: (**A**) Traction-Manipulation in Rest Position C0–C1; C1–C2; C2–C3 (**B**) Interapophisary Traction-Manipulation C2–3.

**Figure 5:**
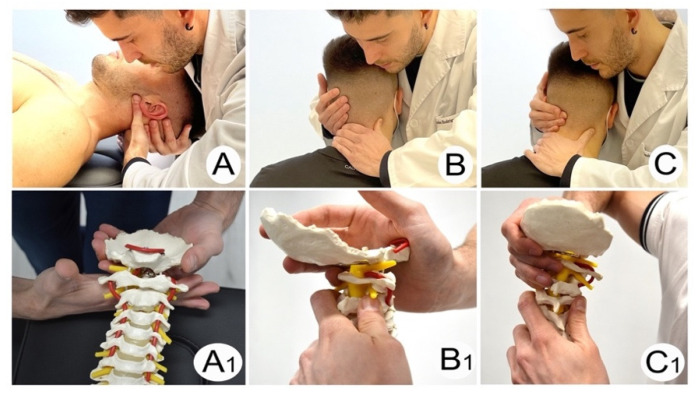
Mobilization Techniques: (**A**,**A1**) Upper Cervical Translatoric Dorsal Glide C0–C1; (**B**,**B1**) Upper Cervical Translatoric Dorsal Glide C1–C2; (**C**,**C1**) Ventral-Cranial Glide C2–C3.

The MT + E group carried out 20 min sessions led by an experienced physical therapist. Manipulation (high-velocity low amplitude) (Figure 4) and/or mobilization (low-velocity high amplitude) (Figure 5) techniques of the upper cervical spine, including the C2–3 segment, were combined with cervical exercise [33,34].

The election of the MT techniques was made depending on the quantity and quality of the loss of mobility, through manual assessment found in each patient. Manual assessment of segmental mobility showed good reliability for the cervical spine (intraclass correlation coefficient between 0.78 and 1) [17]. The segmental assessment included the quantity and quality of traction and gliding in each segment [16]. If the traction was restricted, a manipulation technique was applied. If the gliding was restricted, a mobilization technique was applied. The objective was to restore the function of the upper cervical spine before applying cervical exercises.

The cervical exercise performed by this group followed the same dose as the Exercise group. The only difference was that the rest between each exercise repetition in this group took 30 s. This was done in order to have 3 min of time to apply the manual therapy techniques before the exercise. All the techniques used in this trial follow IFOMPT recommendations to reduce the risk of adverse events [21].

### 2.5. Statistical Analysis

Statistical analysis was conducted with the SPSS 23.0 package (IBM, Armonk, NY, USA). There was a loss of follow-up in the exercise group (Figure 1). The mean and standard deviation were calculated for each variable. The Kolmogorov–Smirnov test was used to determine a normal distribution of quantitative data and the Chi-Square test for sex variables (*p* > 0.05). Intragroup and intergroup differences were analyzed using the Student *t* test. For the variables that did not follow a Gaussian distribution, nonparametric analysis was carried out for statistical evaluation using the Mann–Whitney *U* test and Wilcoxon signed-rank test. Effect sizes were calculated using Cohen’s d coefficient [35]. An effect size > 0.8 was considered large; around 0.5, intermediate; and <0.2, small [35]. All patients enrolled originally were included in the final analysis as planned. Thus, patients were analyzed as per protocol (Little’s missing completely at random test and expectation maximization). The level of significance was set at *p* < 0.05.

## 3. Results

From October 2021 to December 2021, 81 volunteers were recruited. Fifty-eight patients (17 male and 41 female), with a mean age of 49.24 (15.89), satisfied all the eligibility criteria and agreed to participate. Twenty-nine patients were randomly assigned to each group, received the treatment as intended, and were analyzed with respect to the outcome. The patients’ demographic characteristics are summarized in Table 1. There were no significant differences between the two groups (*p* > 0.05) (Table 1).

### 3.1. Cervical Range of Motion

A significant decrease in cervical range of motion was observed immediately after the intervention for the exercise group in flexion (*p* < 0.004), extension (*p* < 0.003), left side-bending (*p* < 0.003), right rotation (*p* < 0.028) and left rotation (*p* < 0.050). Pre–post effect sizes were adverse between −0.03 and −0.38 (Table 2). In contrast, the MT + E group had a significant increase in cervical range of movement between pre- and post-intervention measurements for left side-bending (*p* < 0.001) and upper cervical flexion (*p* < 0.004) (Table 2). Pre–post left side-bending effect size was small (d = 0.40) and upper cervical flexion effect size was intermediate (d = 0.74). The MT + E group experienced significant increases in cervical range of motion as compared with the exercise group in extension (*p* < 0.0014), left side-bending (*p* < 0.006), right rotation (*p* < 0.031), left rotation (*p* < 0.024) and upper cervical flexion (*p* < 0.001) (Table 2). Between-group effect sizes were small to large (0.44 < d < 1.11) after the intervention (Table 2).

### 3.2. Flexion-Rotation Test

A significant decrease in ROM was observed immediately after the intervention for the exercise group in the FRT (right) (*p* < 0.028) and FRT (left) (*p* < 0.022) (Table 3). Pre–post effect sizes were adverse between −0.11 for FRT (right) and −0.09 for FRT (left) (Table 3). For the MT + E group, a significant increase in ROM was observed immediately after the intervention in the FRT (right) (*p* < 0.001) and FRT (left) (*p* < 0.001) (Table 3). Pre–post effect sizes were large between 1.16 for FRT (right) and 1.00 for FRT (left) (Table 3). The MT + E group experienced significant increases in ROM as compared with the exercise group in the FRT (right) (*p* < 0.001) and in the FRT (left) (*p* < 0.001) (Table 3). Between-group effect sizes were large after the intervention between 1.39 FRT (left) and 1.79 FRT (right) (Table 3).

### 3.3. Pressure Pain Threshold

A significant decrease in PPT was observed immediately after the intervention for the exercise group between pre- and post-intervention measurements in the first MCJ (right) (*p* < 0.003), elevator of scapula (right) (*p* < 0.004), C5–6 (right) (*p* < 0.003), suboccipital (right) (*p* < 0.001), first MCJ (left) (*p* < 0.001), trapezius (left) (*p* < 0.001), elevator of scapula (left) (*p* < 0.001), C5–6 (left) (*p* < 0.001), C2–3 (left) (*p* < 0.003) and suboccipital (left) (*p* < 0.001) (Table 4). Pre–post effect sizes were adverse, between −0.19 and −0.42 (Table 4). For the MT + E group there were no statistically significant differences between pre- and post-intervention. The MT + E group experienced significant increases in PPT as compared with the exercise group in C5–6 (right) (*p* < 0.039), C2–3 (right) (*p* < 0.025), suboccipital (right) (*p* < 0.004), trapezius (left) (*p* < 0.034), elevator of scapula (left) (*p* < 0.012), C5–6 (left) (*p* < 0.018), C2–3 (left) (*p* < 0.015) and suboccipital (left) (*p* < 0.022) (Table 4). Between-group effect sizes were intermediate (0.56 < d < 0.74) after the intervention (Table 4).

### 3.4. VAS

For the exercise group, there were no statistically significant differences between pre- and post-intervention in VAS measurement (*p* > 0.965) (Table 5). For the MT + E group, a significant decrease in VAS was observed immediately after the intervention (*p* < 0.001). Pre–post effect size was large (d = 0.92) (Table 5). The MT + E group experienced a significant decrease in VAS as compared with the exercise group (*p* < 0.001). The between-group effect size was large (d = 1.01) after the intervention (Table 5).

## 4. Discussion

### 4.1. Cervical Range of Motion

Loss in cervical ROM in the exercise group is a finding that has not been described until now in any other study on cervical exercise undertaken on patients with chronic neck pain. Other studies that conducted interventions based on MT + E or exercise on deep flexor muscles in an isolated way yielded improvements or no changes in cervical ROM; however, none yielded decreases [36].

One study found that a wide ROM in terms of flexion of the upper cervical spine is needed to be able to make the “chin tuck” maneuver used for exercising the deep neck flexor muscles [12]. There is controversy about the normal ROM of upper cervical flexion. Most authors consider values between 14 and 14.5° to be normal [37]. In our study, both groups showed less upper cervical flexion ROM than normal in the pre-treatment evaluation, but in the post-treatment evaluation, the MT + E group obtained 14.38° ± 3.68°. Thus, in the post-treatment evaluation, the MT + E group obtained upper cervical flexion values considered to be non-pathological [37]. It is possible that the ROM deficit in the upper cervical spine made it difficult to carry out the “chin tuck” maneuver in the exercise group.

Due to the relevant contribution of the upper cervical spine within the cervical movement, the upper cervical dysfunction may impact the cervical ROM [38]. This reason can explain why restoring upper cervical flexion in the MT + E group had an immediate effect on the cervical ROM in terms of left-side bending. However, the measure inaccuracy of the CROM ranges has been established by one study as being between 5 and 10° [29]; thus, these results should be considered with caution.

### 4.2. Flexion-Rotation Test

All patients had a positive FRT at the beginning of the study, but in the post-treatment evaluation, the MT + E group obtained FRT values considered to be non-pathological [18]. The significant improvement in the ROM for the FRT in the MT + E group can be explained by the specific manual treatments received by each patient aiming to restore mobility for specific segments. The FRT measures upper cervical rotation, mainly in the C1–C2 segment. These patients received techniques to restore motion in C1–2 and also in the C0–1 and C2–3 segments. There are studies that have shown improvements in FRT by applying MT to the C1–2 segment [39]. Other studies have presented that, by treating C0–1 or C2–3 segments, the function of the segment C1–2 and in the FRT improved [40,41]. Our results in the MT + E group are better than the results of these previous studies. It is possible that this is due to the fact that in our study, the C1–2 segment also received techniques to restore motion if dysfunction in the mobility was detected. In terms of the decrease in the ROM for the FRT in the exercise group, it can be hypothesized by the relationship between flexion restriction in the upper cervical spine and the rotation of segment C1–2, by which a lack of motion in segment C0–1 would bring about premature tensioning of the alar ligaments and, therefore, decreased motion in segment C1–2 [41]. A recent in vitro study has shown that the stabilization of C0–C1 reduced the upper cervical ROM by 15.6% in the transverse plane [42]. In vitro, with C2 fixated, and after unilateral transection of the alar ligament, a predominantly bilateral increase in upper cervical side bending [43] and a bilateral increase in the upper cervical rotation [44] have been revealed.

### 4.3. Pressure Pain Threshold

We found only one study that assessed the pressure pain threshold immediately with a sample with upper cervical spine dysfunction, though it was conducted in patients with cervicogenic headaches. The author did not find significant differences between the control group and the group that received MT treatment (C0–C1 translatoric mobilization) in the upper trapezius muscle, splenius cervicis and suboccipital muscles [40]. The higher sensitivity of the exercise group at most of the pressure points may be due to tissue irritation processes because of exercising deep cervical muscles through upper cervical spine flexion without a proper ROM. Because this chronic neck pain subgroup has a dysfunction specific to that region and motion, the irritation could affect central processes produced by activation of segmental inhibitory pathways, spinal cord pathways, or descending inhibitory pathways from the brainstem [45], which would result in the higher sensitivity of the distant points, such as the first MCJ (R) and (L).

### 4.4. VAS

The exercise group did not experience significant differences in pain despite the slight increase, whereas the MT + E group experienced a significant decrease in pain.

The hypoalgesic effect of MT and exercise has been explained based on an integrated approach. Bialosky JE et al. (2009) defended a model based on a mechanical stimulus initiating a chain of spinal neurophysiological events, peripheral events and/or supraspinal events that would produce this hypoalgesia. Nevertheless, not all manual techniques would produce similar neurophysiological effects; instead, this would depend on the stimulus and the dose [46].

### 4.5. Clinical Implications

Chronic pain is a complex clinic entity whose therapeutic approach may require multimodal treatment protocols to improve effectiveness. Previous studies have shown the effectiveness of exercise when combined with other therapies for symptom treatment in patients with chronic low back pain [47] and the multimodal approaches have been preconized in nonspecific neck pain [48]. The current study showed that patients with mobility restrictions in the upper cervical spine may benefit more from therapeutic exercise if the mobility restrictions had previously been treated with manual therapy.

### 4.6. Limitations

The main limitation of this study is the nature of the intervention. To undertake interventions more clinical in nature renders it impractical to know which specific intervention has had the greatest impact on the patients. Another important limitation is that mechanical neck pain can be caused by visceral disorders. In this study, the visceral origin was not considered as an exclusion criterium [49]. Due to the fact that some of the changes in the cervical range of motion are small and may be due to measurement inaccuracy, these data should be taken with caution. However, the changes in the FRT are sufficiently broad and consistent. Finally, the small sample size coming from a single population nucleus can compromise the generalization of the results to other populations.

This study provides only immediate effects; however, if the reader wants to know the short- and medium-term effects, in a similar subgroup of patients, the following publications can be consulted [13,19]. It would be necessary to undertake new studies with a long-term scope to identify whether the findings obtained for immediate effects remain the same for the new subgroup of patients with chronic neck pain and upper cervical spine dysfunction, treated with manual therapy in C0–C1, C1–C2 and C2–C3 segments.

## 5. Conclusions

This study showed that an isolated exercise session produced a significant immediate decrease in the ROM, FRT and PPT variables for the subgroup of patients with chronic neck pain and mobility deficits in the upper cervical spine. A specific treatment session with MT, on C0–1, C1–2 and C2–3 segments, along with exercise showed significant improvements in the upper cervical ROM, FRT and VAS when compared with the exercise group. It may be necessary to normalize the mobility in the upper cervical spine before cervical stabilization training, in patients with chronic neck pain and mobility deficits in the upper cervical spine.

## Figures and Tables

**Table 1:** Baseline Features for Both Groups.

	E Group (*n* = 29)	MT + E Group (*n* = 29)	*p* Value
**Clinical Features**			
Age (years)	49.72 ± 17.56	48.76 ± 14.53	0.820 ^t^
Sex	7 M; 22 F	10 M; 19 F	0.565 ^C^
Duration of Symptoms (months)	124.38 ± 148.17	96.97 ± 96.73	0.502 ^M^
**Current Pain Intensity** (mm)	37.55 ± 25.32	33.62 ± 19.70	0.512 ^t^
**Cervical ROM** (°)			
Cervical Flexion	48.10 ± 10.93	47.48 ± 12.85	0.844 ^t^
Cervical Extension	51.48 ± 12.66	53.59 ± 14.36	0.557 ^t^
Cervical Side-bending (R)	27.97 ± 8.59	32.03 ± 9.93	0.101 ^t^
Cervical Side-bending (L)	29.38 ± 9.12	30.28 ± 9.83	0.720 ^t^
Cervical Rotation (R)	53.97 ± 12.32	55.66 ± 16.07	0.429 ^M^
Cervical Rotation (L)	55.28 ± 14.34	58.14 ± 16.37	0.482 ^t^
Upper Cervical Flexion	10.59 ± 4.39	11.45 ± 4.24	0.450 ^t^
Upper Cervical Extension	24.14 ± 8.12	28.28 ± 7.56	0.070 ^M^
**FRT ROM** (°)			
FRT (R)	16.70 ± 9.52	21.26 ± 10.71	0.092 ^t^
FRT (L)	19.01 ± 10.33	23.12 ± 8.98	0.094 ^M^
**Pressure Pain Threshold** (Kpa)			
First MCJ (R)	359.14 ± 175.98	395.93 ± 195.23	0.504 ^M^
Trapezius (R)	192.17 ± 88.42	208.00 ± 98.75	0.523 ^t^
Elevator of Scapula (R)	180.69 ± 105.62	213.45 ± 132.29	0.259 ^M^
C5–6 (R)	152.86 ± 63.17	177.59 ± 84.66	0.39 ^M^
C2–3 (R)	173.76 ± 87.92	208.69 ± 114.53	0.347 ^M^
Suboccipital (R)	186.10 ± 75.34	211.45 ± 91.57	0.255 ^t^
First MCJ (L)	364.34 ± 155.47	339.90 ± 184.74	0.222 ^M^
Trapezius (L)	213.28 ± 97.49	237.97 ± 113.66	0.437 ^M^
Elevator of Scapula (L)	190.24 ± 122.36	223.62 ± 141.34	0.287 ^M^
C5–6 (L)	153.90 ± 72.56	175.76 ± 76.25	0.253 ^M^
C2–3 (L)	174.59 ± 90.02	206.38 ± 113.72	0.256 ^M^
Suboccipital (L)	180.59 ± 79.85	207.90 ± 105.33	0.494 ^M^

^t^. T-Student; ^C^. Chi-Square Test; ^M^. Mann–Whitney *U* test; M. male; F. female; ROM. range of motion; FRT. flexion-rotation test; R. right; L. left; MCJ. metacarpal joint; E. Exercise; MT + E. Manual Therapy and Exercise.

**Table 2:** Pre- and Post-Treatment in Cervical Range of Motion Outcomes.

Outcome/Group	Pre-Treatment	Post-Treatment	Within-Group	Between-Group
**Cervical Flexion** (°)				
E Group	48.10 ± 10.93	44.04 ± 10.65	*p* < 0.004 *^↓^^W^	*p* = 0.167 ^t^ d = 0.37
			d = −0.38	
MT + E Group	47.48 ± 12.85	48.03 ± 10.89	*p* = 0.759 ^t^	
			d = 0.05	
**Cervical Extension** (°)				
E Group	51.48 ± 12.66	48.71 ± 10.25	*p* < 0.003 *^↓^^t^	*p* < 0.014 *^t^ d = 0.68
			d = −0.24	
MT + E Group	53.59 ± 14.36	56.38 ± 12.34	*p* = 0.064 ^t^	
			d = 0.21	
**Cervical Side-Bending (R)** (°)				
E Group	27.97 ± 8.59	27.71 ± 8.29	*p* = 0.152 ^t^	*p* = 0.109 ^M^ d = 0.50
			d = −0.03	
MT + E Group	32.03 ± 9.93	31.90 ± 8.43	*p* = 0.910 ^t^	
			d = −0.01	
**Cervical Side-Bending (L)** (°)				
E Group	29.38 ± 9.12	27.61 ± 8.51	*p* < 0.003 *^↓^^t^	*p* < 0.006 *^t^ d = 0.76
			d = −0.20	
MT + E Group	30.28 ± 9.83	33.83 ± 7.88	*p* < 0.001 *^↑^^t^	
			d = 0.40	
**Cervical Rotation (R)** (°)				
E Group	53.97 ± 12.32	52.54 ± 12.13	*p* < 0.028 *^↓^^W^	*p* < 0.031 *^M^ d = 0.44
			d = −0.12	
MT + E Group	55.66 ± 16.07	58.24 ± 13.97	*p* = 0.508 ^W^	
			d = 0.17	
**Cervical Rotation (L)** (°)				
E Group	55.28 ± 14.34	53.43 ± 13.43	*p* < 0.050 *^↓^^t^	*p* < 0.024 *^t^ d = 0.62
			d = −0.13	
MT + E Group	58.14 ± 16.37	61.76 ± 13.58	*p* = 0.135 ^t^	
			d = 0.24	
**Upper Cervical Flexion** (°)				
E Group	10.59 ± 4.39	10.18 ± 3.92	*p* = 0.471 ^t^	*p* < 0.001 *^t^ d = 1.11
			d = −0.10	
MT + E Group	11.45 ± 4.24	14.38 ± 3.68	*p* < 0.004 *^↑^^t^	
			d = 0.74	
**Upper Cervical Extension** (°)				
E Group	23.14 ± 8.12	22.57 ± 8.46	*p* = 0.492 ^t^	*p* = 0.627 ^M^ d = 0.45
			d = −0.07	
MT + E Group	29.28 ± 7.56	27.10 ± 11.35	*p* = 0.265 ^W^	
			d = −0.23	

^t^. T-Student; ^W^. Wilcoxon signed-rank test; ^M^. Mann–Whitney *U* test; R. right; L. left; E. Exercise; MT + E. Manual Therapy + Exercise; (*). statistically significant differences; (^↓^). decrease;(^↑^). increase.

**Table 3:** Pre- and Post-Treatment in FRT Range of Motion Outcomes.

Outcome/Group	Pre-Treatment	Post-Treatment	Within-Group	Between-Group
**FRT (R)** (°)				
E Group	16.70 ± 9.52	15.61 ± 10.03	*p* < 0.028 *^↓^^W^	*p* < 0.001 *^t^ d = 1.79
			d = −0.11	
MT + E Group	21.26 ± 10.71	32.91 ± 9.29	*p* < 0.001 *^↑^^W^	
			d = 1.16	
**FRT (L)** (°)				
E Group	19.01 ± 10.33	18.04 ± 11.04	*p* < 0.022 *^↓^^W^	*p* < 0.001 *^M^ d = 1.39
			d = −0.09	
MT + E Group	23.12 ± 8.98	32.26 ± 9.32	*p* < 0.001 *^↑^^W^	
			d = 1.00	

^t^. T-Student; ^W^. Wilcoxon signed-rank test; ^M^. Mann–Whitney *U* test; FRT. flexion-rotation test; R. right; L. left; E. Exercise; MT + E. Manual Therapy + Exercise; (*). statistically significant differences; (^↓^). decrease; (^↑^). increase.

**Table 4:** Pre- and Post-Treatment in PPT Outcomes.

Outcome/Group	Pre-Treatment	Post-Treatment	Within-Group	Between-Group
**First MCJ (R)** (Kpa)				
E Group	359.14 ± 175.98	325.64 ± 166.49	*p* < 0.003 *^↓^^t^	*p* = 0.190 ^M^ d = 0.34
			d = −0.20	
MT + E Group	395.93 ± 195.23	385.62 ± 188.31	*p* = 0.658 ^W^	
			d = −0.05	
**Trapezius (R)** (Kpa)				
E Group	192.17 ± 88.42	183.86 ± 91.02	*p* = 0.278 ^t^	*p* = 0.225 ^M^ d = 0.31
			d = −0.09	
MT + E Group	208 ± 98.75	213.07 ± 99.04	*p* = 0.646 ^t^	
			d = 0.05	
**Elevator of Scapula (R)** (Kpa)				
E Group	180.69 ± 105.62	162.64 ± 84.42	*p* < 0.004 *^↓^^W^	*p* = 0.076 ^t^ d = 0.48
			d = −0.19	
MT + E Group	213.45 ± 132.29	212.1 ± 118.62	*p* = 0.585 ^W^	
			d = −0.01	
**C5–6 (R)** (Kpa)				
E Group	152.86 ± 63.17	138.32 ± 60.34	*p* < 0.003 *^↓^^t^	*p* < 0.039 *^t^ d = 0.56
			d = −0.24	
MT + E Group	177.59 ± 84.66	179.76 ± 85.29	*p* = 0.405 ^W^	
			d = 0.03	
**C2–3 (R)** (Kpa)				
E Group	173.76 ± 87.92	161.36 ± 78.84	*p* = 0.076 ^W^	*p* < 0.025 *^M^ d = 0.59
			d = −0.15	
MT + E Group	208.69 ± 114.53	221.79 ± 119.76	*p* = 0.336 ^W^	
			d = 0.11	
**Suboccipital (R)** (Kpa)				
E Group	186.1 ± 75.34	153.25 ± 79.66	*p* < 0.001 *^↓^^W^	*p* < 0.004 *^M^ d = 0.74
			d = −0.42	
MT + E Group	211.45 ± 91.57	227.41 ± 117.72	*p* = 0.193 ^t^	
			d = 0.15	
**First MCJ (L)** (Kpa)				
E Group	364.34 ± 155.47	300.96 ± 142.8	*p* < 0.001 *^↓^^t^	*p* = 0.384 ^M^ d = 0.28
			d = −0.42	
MT + E Group	339.9 ± 184.74	342.55 ± 151.68	*p* = 0.658 ^W^	
			d = 0.02	
**Trapezius (L)** (Kpa)				
E Group	213.28 ± 97.49	177.96 ± 89.33	*p* < 0.001 *^↓^^W^	*p* < 0.034 *^t^ d = 0.58
			d = −0.38	
MT + E Group	237.97 ± 113.66	234.31 ± 105.56	*p* = 0.698 ^t^	
			d = −0.03	
**Elevator of Scapula (L)** (Kpa)				
E Group	190.24 ± 122.36	157.25 ± 83.64	*p* < 0.001 *^↓^^W^	*p* < 0.012 *^M^ d = 0.66
			d = −0.31	
MT + E Group	223.62 ± 141.34	222.76 ± 112.67	*p* = 0.509 ^W^	
			d = −0.01	
**C5–6 (L)** (Kpa)				
E Group	153.9 ± 72.56	130.89 ± 65.49	*p* < 0.001 *^↓^^t^	*p* < 0.018 *^t^ d = 0.65
			d = −0.33	
MT + E Group	175.76 ± 76.25	175.34 ± 71.71	*p* = 0.871 ^W^	
			d = −0.01	
**C2–3 (L)** (Kpa)				
E Group	174.59 ± 90.02	146.54 ± 78.57	*p* < 0.003 *^↓^^t^	*p* < 0.015 *^t^ d = 0.67
			d = −0.33	
MT + E Group	206.38 ± 113.72	208.24 ± 103.84	*p* = 0.665 ^W^	
			d = 0.02	
**Suboccipital (L)** (Kpa)				
E Group	180.59 ± 79.85	152.46 ± 70.75	*p* < 0.001 *^↓^^t^	*p* < 0.022 *^M^ d = 0.68
			d = −0.37	
MT + E Group	207.9 ± 105.33	214.38 ± 107.5	*p* = 0.456 ^W^	
			d = 0.06	

^t^. T-Student; ^W^. Wilcoxon signed-rank test; ^M^. Mann–Whitney *U* test; R. right; L. left; MCJ. metacarpal joint; E. Exercise; MT + E. Manual Therapy + Exercise; (*). statistically significant differences; (^↓^). Decrease.

**Table 5:** Pre- and Post-Treatment in Pain Outcomes.

Outcome/Group	Pre-Treatment	Post-Treatment	Within-Group	Between-Group
**EVA** (mm)				
E Group	37.55 ± 25.32	37.9 ± 24.3	*p* = 0.965 ^t^	*p* < 0.001 *^M^ d = 1.01
			d = −0.01	
MT + E Group	33.62 ± 19.70	16.0 ± 18.6	*p* < 0.001 *^↑^^w^	
			d = 0.92	

^t^. T-Student; ^W^. Wilcoxon signed-rank test; ^M^. Mann–Whitney *U* test; E. Exercise; MT + E. Manual Therapy + Exercise; (*). statistically significant differences; (^↑^). Less pain.

## Data Availability

The datasets analyzed during the current study are available from the corresponding author on reasonable request. All data analyzed during this study are included in this published article.
